# Differential effects of winter cold stress on soil bacterial communities, metabolites, and physicochemical properties in two varieties of *Tetrastigma hemsleyanum* Diels & Gilg in reclaimed land

**DOI:** 10.1128/spectrum.02425-23

**Published:** 2024-03-12

**Authors:** Xuqing Li, Xiaoxu Ren, Yao Su, Xiang Zhou, Yu Wang, Songlin Ruan, Jianli Yan, Bin Li, Kai Guo

**Affiliations:** 1 Institute of Vegetable, Hangzhou Academy of Agricultural Sciences, Hangzhou, China; 2 Institute of Environment, Resource, Soil and Fertilizer, Zhejiang Academy of Agricultural Sciences, Hangzhou, China; 3 Hangzhou Agricultural and Rural Affairs Guarantee Center, Hangzhou, China; 4 Qingliangfeng Lvyuan Vegetable Professional Cooperative, Hangzhou, China; 5 Institute of Biotechnology, Zhejiang University, Hangzhou, China; 6 School of Forestry and Biotechnology, Zhejiang A&F University, Hangzhou, China; University of Mississippi, University, Mississippi, USA

**Keywords:** *Tetrastigma hemsleyanum*, Diels & Gilg (TDG), winter cold stress, reclaimed land, 16S rRNA, soil bacterial communities, soil metabolites, soil properties

## Abstract

*Tetrastigma hemsleyanum* Diels & Gilg (TDG) has been recently planted in reclaimed lands in Zhejiang Province, China, to increase reclaimed land use. Winter cold stress seriously limits the growth and development of TDG and has become the bottleneck limiting the TDG planting industry. To investigate the defense mechanisms of TDG toward winter cold stress when grown on reclaimed land, a combined analysis of soil bacterial communities, metabolites, and physicochemical properties was conducted in this study. Significant differences were observed in the composition of soil bacterial communities, metabolites, and properties in soils of a cold-tolerant variety (A201201) compared with a cold-intolerant variety (B201810). The fresh weight (75.8% of tubers) and dry weight (73.6%) of A201201 were significantly higher than those of B201810. The 16S rRNA gene amplicon sequencing of soil bacteria showed that Gp5 (25.3%), *Gemmatimonas* (19.6%), Subdivision3 (16.7%), *Lacibacterium* (11.9%), Gp4 (11.8%), Gp3 (10.4%), Gp6 (7.0%), and WPS-1 (1.2%) were less common, while *Chryseolinea* (10.6%) were more common in A201201 soils than B201810 soils. Furthermore, linear discriminant analysis of effect size identified 35 bacterial biomarker taxa for both treatments. Co-occurrence network analyses also showed that the structures of the bacterial communities were more complex and stable in A201201 soils compared to B201810 soils. In addition, ultra-high-performance liquid chromatography coupled to mass spectrometry analysis indicated the presence of significantly different metabolites in the two soil treatments, with 10 differentially expressed metabolites (DEMs) (8 significantly upregulated by 9.2%–391.3% and 2 significantly downregulated by 25.1%–73.4%) that belonged to lipids and lipid-like molecules, organic acids and derivatives, and benzenoids. The levels of those DEMs were significantly correlated with the relative abundances of nine bacterial genera. Also, redundancy discriminant analysis revealed that the main factors affecting changes in the bacterial community composition were available potassium (AK), microbial biomass nitrogen (MBN), microbial biomass carbon (MBC), alkaline hydrolysis nitrogen (AHN), total nitrogen (TN), available phosphorus (AP), and soil organic matter (SOM). The main factors affecting changes in the metabolite profiles were AK, MBC, MBN, AHN, pH, SOM, TN, and AP. Overall, this study provides new insights into the TDG defense mechanisms involved in winter cold stress responses when grown on reclaimed land and practical guidelines for achieving optimal TDG production.

**IMPORTANCE:**

China has been undergoing rapid urbanization, and land reclamation is regarded as a viable option to balance occupation and compensation. In general, the quality of reclaimed land cannot meet plant or even cultivation requirements due to poor soil fertility and high gravel content. However, *Tetrastigma hemsleyanum* Diels & Gilg (TDG), extensively used in Chinese herbal medicine, can grow well in stony soils with few nutrients. So, to increase reclaimed land use, TDG has been cultivated on reclaimed lands in Zhejiang Province, China, recently. However, the artificial cultivation of TDG is often limited by winter cold stress. The aim of this study was to find out how TDG on reclaimed land deal with winter cold stress by looking at the bacterial communities, metabolites, and physicochemical properties of the soil, thereby guiding production in practice.

## INTRODUCTION

*Tetrastigma hemsleyanum* Diels & Gilg (TDG), also known as Sanyeqing in China, is a perennial climbing liana plant in the Vitaceae family (1, 2). Sanyeqing is specific to China and primarily distributed in tropical to subtropical regions, including in the Zhejiang, Fujian, Guangxi, Jiangsu, and Jiangxi provinces (1, 3). The plants have flavonoids, amino acids, and terpenoids in them (1, 4). The flavonoids are the active ingredients that have properties that directly affect inflammation, pain, fever, tumors, viruses, and immune system regulation (1, 5, 6). The tuberous roots and whole grass plant components have been widely used in traditional Chinese medicine for treating diseases including high fevers (especially febrile convulsions in children), pneumonia, snake bites, cellulitis, asthma, hepatitis, gastritis, cervicitis, lymphatic tuberculosis, septicemia, viral meningitis, and cancers (2, 5, 7). However, its widespread use in many Chinese drugs and health products, including Huatuofengtongbao, Jinsidijia, and Jieshikang capsules (8), in addition to Huashixuanfeiheji developed in the Zhejiang Province to mitigate common COVID-19 (5, 9), has led to TDG being on the verge of extinction due to overexploitation (10). Therefore, the improved artificial cultivation of TDG is urgently needed to reduce dependence on wild resources (11). TDG has been recently cultivated on reclaimed lands in Zhejiang Province to increase reclaimed land use. Reclaimed land is typically acidic and comprises poor nutrient levels and a high gravel content (12), leading to suboptimal conditions for typical plant growth. However, TDG has been shown to grow in stony and poor soils with better-developed root systems and higher yields (13, 14). Nevertheless, due to poor cold resistance, cold temperatures in winter have become a major stressor, markedly restricting the artificial cultivation of TDG in reclaimed land, thereby seriously limiting the development of TDG (11). Specifically, cold stress can not only significantly affect the growth of TDG but also have significant effects on a series of physiological and biochemical processes (such as free radical burst, osmotic stress, and material metabolism disorder in cells), which can seriously affect the quality of TDG (11).

Cold stress is a primary environmental factor affecting plant growth, development, and geographical distribution (15, 16). Low temperatures mainly disrupt the critical processes of seed germination and seedling establishment (17), limit CO_2_ fixation, decrease photosynthetic capacity, increase reactive oxygen species (18), and cause serious damage to yield formation (19). To resist abiotic stress, organisms have developed defense mechanisms over their long evolutionary histories. Waadt et al. (20) showed that plant hormones are signaling compounds that regulate crucial aspects of growth, development, and environmental stress responses. Abbasi et al. (21) further demonstrated that fungal and bacterial communities were resistant to drought stress. Barria et al. (22) also said that changes in RNA metabolism, such as the specific induction of RNA chaperones and changes in the levels of certain RNases, help single-celled organisms survive cold temperatures. Likewise, Peng et al. (4) documented transcriptomic changes in response to cold stress. Previous studies have also shown that soil microorganisms are important components of agriculture ecosystems because microbial communities can help maintain soil fertility by transforming, solubilizing, and mobilizing soil nutrients. Furthermore, soil microbial communities promote plant growth and soil metabolites, thereby playing an important role in rhizosphere soil environments by facilitating plant-microbe interactions (23–26). Nevertheless, additional research is needed to identify relationships among soil properties, microorganisms, metabolites, and plants from multiple perspectives.

Here, we hypothesized that soil-associated bacterial communities and metabolites play critical roles in determining TDG fitness for winter cold stress within TDG plantations on reclaimed land. The goal of this study was to use a combined analysis of soil bacterial communities, metabolites, and properties to identify TDG mechanisms involved in winter cold stress responses within reclaimed land. The results provide a scientific framework for understanding the tolerance of TDG to winter cold stress.

## RESULTS

### A201201 and B201810 tuber biomass and quality after winter cold stress

**Fig 1:**
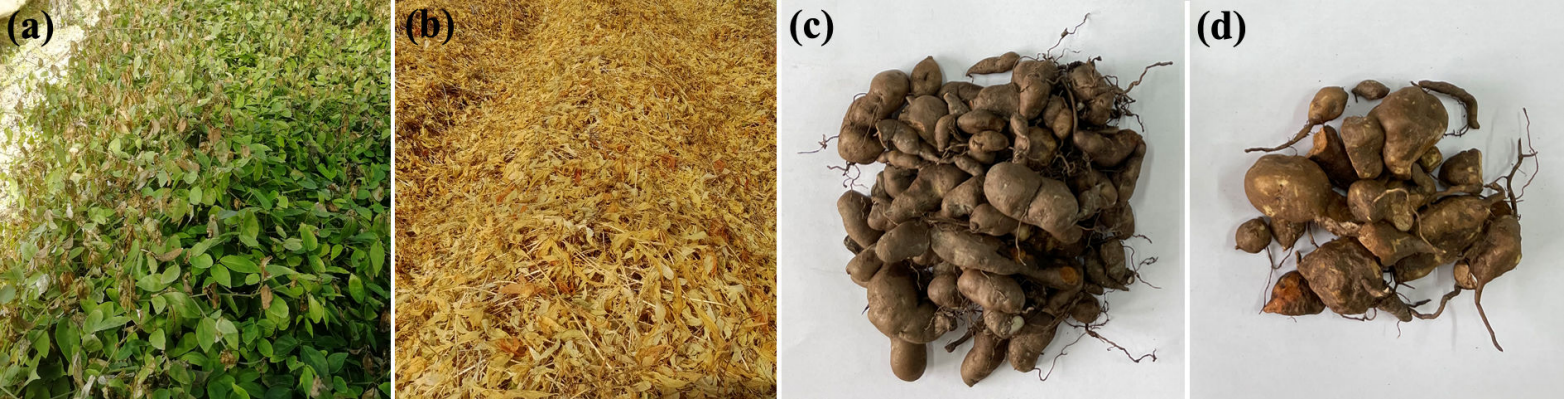
Image of the experimental field plot and tuber biomass of A201201 (**a and c**) and B201810 (**b and d**) grown on reclaimed land.

The fresh and dry weights of tubers were measured about 42 months after planting to estimate the impact of winter cold stress on the tuber biomass of TDG in reclaimed lands. Winter cold stress occurred over 3 months every year (from December to February), leading to the aboveground components (including leaves and stems) of B201810 turning yellow after wilting, while those of A201201 remained green (Fig. 1a and b). Significant differences in tuber biomass between A201201 and B201810 were also observed (Fig. 1c and d; Table 1). Specifically, 75.8% and 73.6% increases in the fresh and dry weights of tubers in A201201 were observed, respectively, relative to those of B201810. In addition, the lengths of tubers in A201201 were 1.4-fold greater than those in B201810, while the widths were not dramatically different between the two. The total flavonoid content (TFC) of A201201 was 0.8-fold lower than that of B201810. Thus, after winter cold stress, A201201 and B201810 growth significantly differed, and the TFC showed a reverse trend to the yield (the higher the yield, the lower the TFC).

**TABLE 1:** Tuber biomass and quality after winter cold stress^*a*^

Parameter	Value	Parameter	Value
Fresh weight (g)		Dry weight (g)	
A201201	120.68 ± 3.71 *	A201201	26.74 ± 1.49 *
B201810	68.63 ± 4.21	B201810	15.40 ± 1.37
Length (mm)		Width (mm)	
A201201	39.14 ± 3.46 *	A201201	16.28 ± 1.66
B201810	27.47 ± 3.66	B201810	17.13 ± 2.12
Total flavonoid content (%)			
A201201	3.72 ± 0.28 #		
B201810	4.64 ± 0.24		

^*a*^ * and # indicate statistically significant increases or decreases, respectively, compared to B201810 (*P* < 0.05).

### Soil bacterial diversity and community composition after winter cold stress

**Fig 2:**
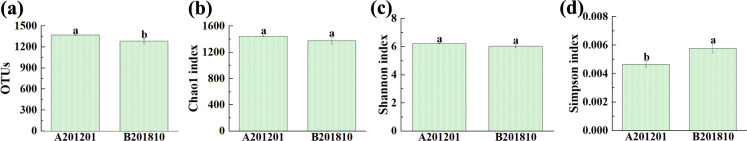
Operational taxonomic unit abundances (**a**) and alpha diversity index levels (b, Chao1; c, Shannon; d, Simpson) of *Tetrastigma hemsleyanum* Diels & Gilg root-zone soil bacterial communities based on OTUs.

A total of 327,649 16S rRNA gene sequences were obtained by high-throughput amplicon sequencing of soil samples, with 161,566 sequences (49.31%) remaining after quality control (24,049–29,992 sequences per sample). A total of 7,964 bacterial operational taxonomic units (OTUs) were identified (Fig. 2a), with an average of 1,371 (1,366–1,375) and 1,284 (1,188–1,344) OTUs identified in A201201 and B201810, respectively. The Chao1, Shannon, and Simpson indices were calculated to assess community richness and alpha diversity (Fig. 2b through d). The average Chao1 richness was 1,448 (1,437–1,462) and 1,375 (1,274–1,425), Shannon index values were 6.23 (6.20–6.25) and 6.03 (5.92–6.10), and Simpson index values were 0.005 (0.004–0.005) and 0.006 (0.005–0.006) in the A201201 and B201810 communities, respectively. As a result, the number of bacterial OTUs was significantly higher (6.7%) in the A201201 communities, along with higher Chao1 and Shannon index values (5.3%, 3.2%) but lower Simpson index values (19.2%) than in the B201810 communities.

**Fig 3:**
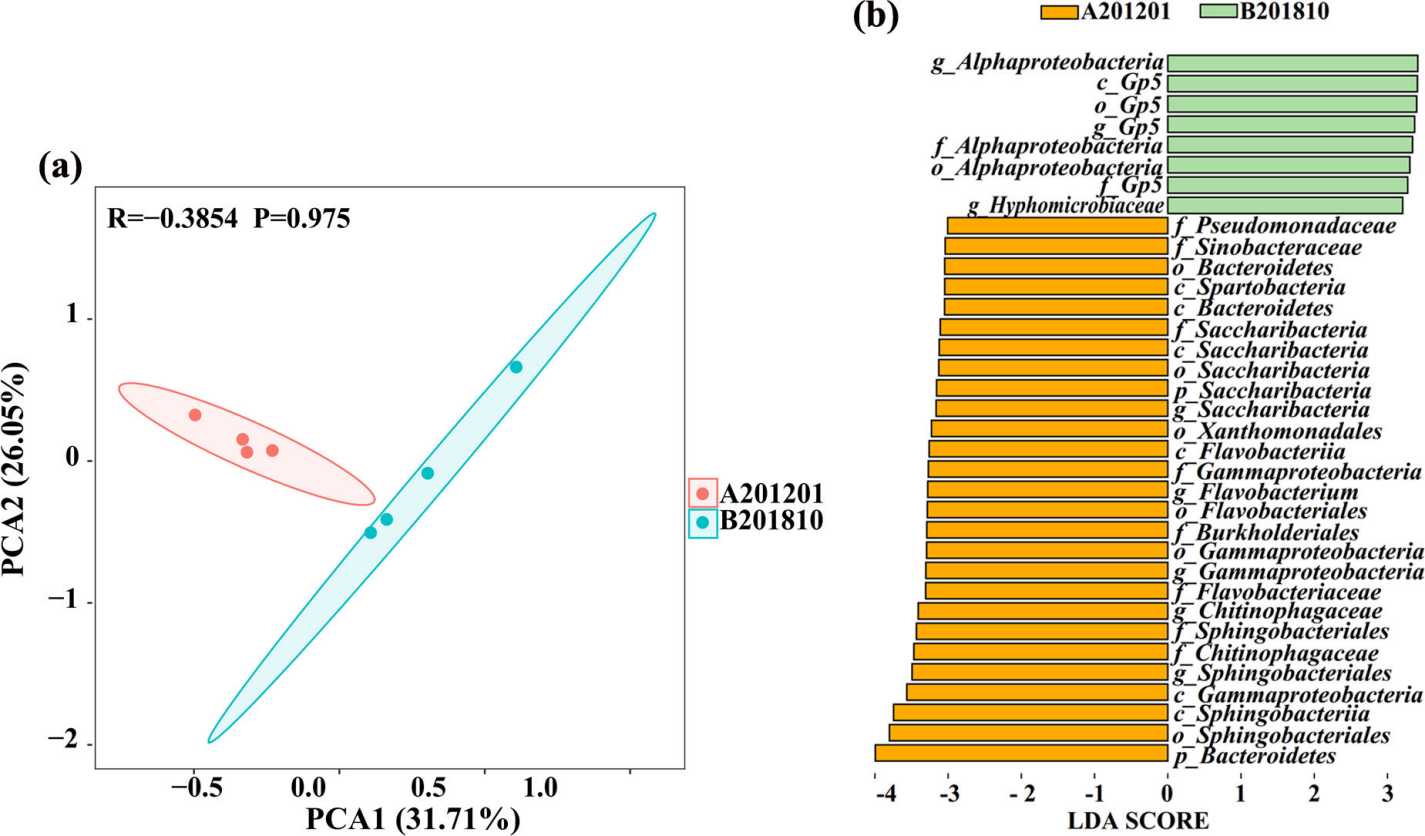
Principal component analysis of *Tetrastigma hemsleyanum* Diels & Gilg root-zone bacterial communities based on compositional variation in OTU abundances (**a**). Ellipses are shown for sample groups when considering a confidence limit of 0.95. Linear discriminant analysis (LDA) effect size evaluation of bacterial taxa revealed the most differentially abundant taxa between A201201 and B201810 soil communities (**b**). Only bacterial taxa with LDA >3 (*P* < 0.05) are shown.

Principal component analysis (PCA) was used to further evaluate bacterial community compositional differences at the OTU level (Fig. 3a). PCA revealed that the soil root-zone bacterial communities of A201201 and B201810 comprised two different groups that were well separated from each other. The first and second principal components (PCA1 and PCA2, respectively) explained 31.71% and 26.05% of the variability in bacterial community composition, respectively. Thus, to a certain extent, the bacterial community structures of A201201 were from those of B201810 after winter cold stress.

**Fig 4:**
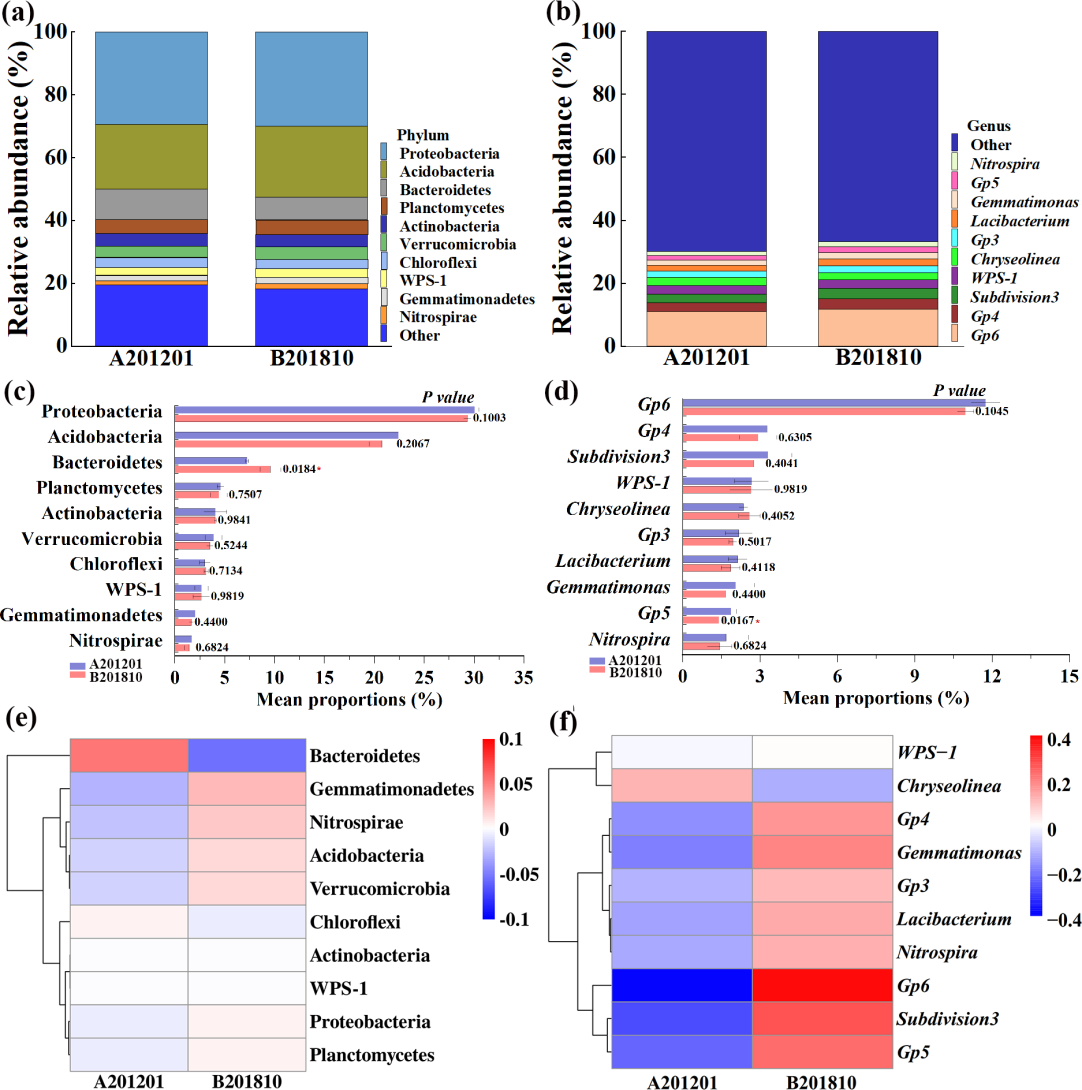
Taxonomic compositions of bacterial communities in A201201 and B201810 soils at the phylum (**a**) and genus (**b**) levels. Analysis of variance between A201201 and B201810 at bacterial phylum (**c**) and genus (**d**) levels. Hierarchical clustering analysis and heatmaps showing phylum (**e**) and genus (**f**) level taxonomic compositions. The clustergrams show variation in the 10 most abundant phyla and genera based on Pearson’s correlation coefficients of relative abundances.

The compositional differences in soil bacterial communities between the A201201 and B201810 groups were compared at the phylum and genus levels. A total of 12 phyla and 26 genera were identified. The relative abundances (RAs) of the 10 most abundant bacterial phyla and genera across all soil samples were particularly evaluated (Fig. 4a and b). Significant differences in the taxonomic composition of bacterial communities were observed when comparing A201201 and B201810 soils. The Proteobacteria, Acidobacteria, Bacteroidetes, Planctomycetes, Actinobacteria, Verrucomicrobia, Chloroflexi, WPS-1, and Gemmatimonadetes were the predominant bacterial phyla, with RAs of 28.95%–30.39%, 19.35%–23.93%, 7.11%–10.80%, 3.71%–5.35%, 2.84%–5.11%, 3.19%–4.85%, 2.46%–3.51%, 1.72%–3.30%, and 1.41%–2.89%, respectively (Fig. 4a). The 10 most abundant genera included the Gp6, Gp4, Subdivision3, WPS-1, *Chryseolinea*, Gp3, *Lacibacterium*, *Gemmatimonas*, Gp5*,* and *Nitrospira*, with RAs of 10.69%–12.12%, 2.34%–4.26%, 2.34%–4.38%, 1.72%–3.30%, 2.21%–3.04%, 1.75%–2.76%, 1.61%–2.49%, 1.41%–2.89%, 1.39%–2.10%, and 0.76%–2.48%, respectively. In A201201 communities, the RAs of Gp5, *Gemmatimonas*, Subdivision3, *Nitrospira*, *Lacibacterium*, Gp4, Gp3, Gp6, and WPS-1 were lower by 25.3%, 19.6%, 16.7%, 14.6%, 11.9%, 11.8%, 10.4%, 7.0%, and 1.2%, while the RA of *Chryseolinea* was higher by 10.6% than that of B201810 (Fig. 4b). Significant changes were consequently observed in the composition of bacterial taxa when comparing the A201201 and B201810 communities. Analysis of variance (ANOVA) and heatmaps were used to visualize differences in RAs of root-zone bacterial communities at the phylum and genus levels (Fig. 4c through f). A201201 communities were enriched with Bacteroidetes (*P* < 0.05) and Chloroflexi but exhibited decreased RAs of Gemmatimonadetes, Nitrospirae, Acidobacteria, and Verrucomicrobia at the phylum level. In addition, A201201 communities were enriched with *Chryseolinea* but exhibited decreased RAs of the genera Gp5 (*P* < 0.05), Gp4, *Gemmatimonas*, Gp6, and Subdivision3 at the genus level. Some changes in the taxonomic compositions of bacterial communities in root-zone soils were thus observed overall when comparing A201201 and B201810 soils after winter cold stress.

To identify taxa enriched in the A201201 and B201810 communities, a linear discriminant analysis (LDA) of effect size (LEfSe; LDA >3, *P* < 0.05) analysis was conducted (Fig. 3b). A total of 35 biomarker taxa were identified for either A201201 or B201810 soil bacterial communities. A201201 communities were enriched with Pseudomonadaceae, Sinobacteraceae, three types of Bacteroidetes, Spartobacteria, five types of Saccharibacteria, Xanthomonadales, Flavobacteriia, four types of Gammaproteobacteria, *Flavobacterium*, Flavobacteriales, Burkholderiales, Flavobacteriaceae, two types of Chitinophagaceae, three types of Sphingobacteriales, and Sphingobacteriia. B201810 communities were enriched with three types of Alphaproteobacteria, four types of Gp5, and Hyphomicrobiaceae. These bacterial taxa may therefore play an important role in modulating TDG growth, particularly when faced with abiotic stresses such as cold winter temperatures.

### Co-occurrence networks of A201201 and B201810 soil bacterial communities

**Fig 5:**
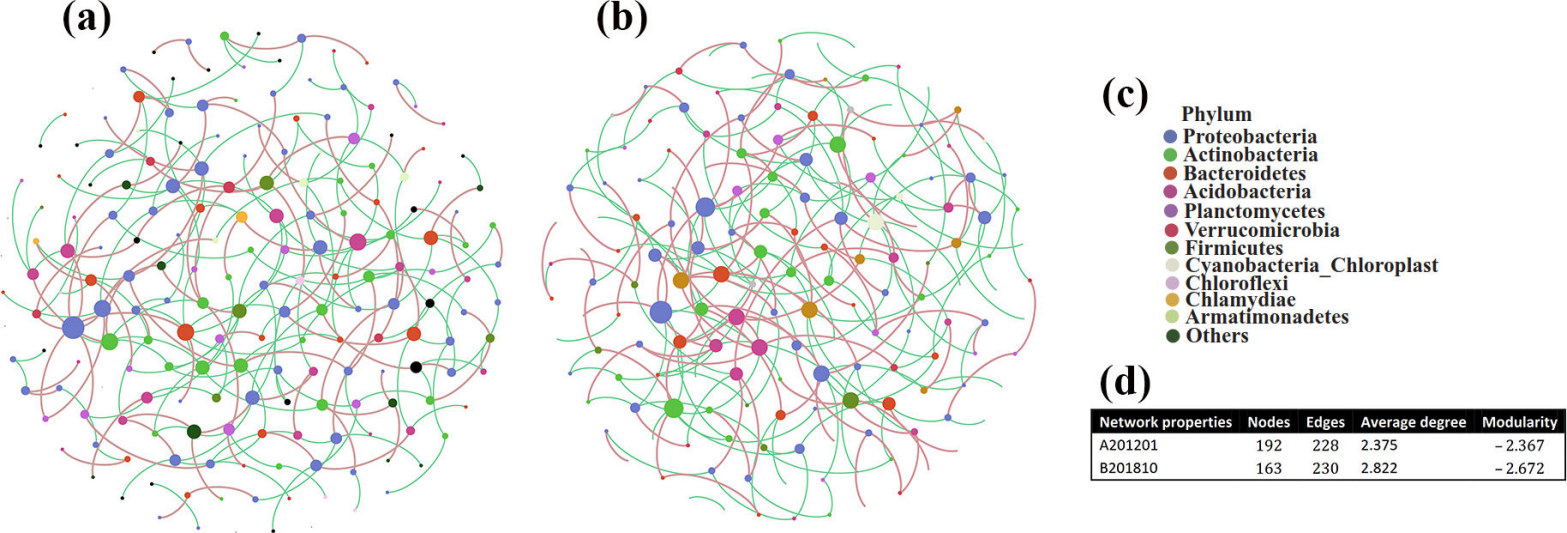
Co-occurrence patterns after cold stress within soil bacterial communities A201201 (**a**) and B201810 (**b**). Networks were constructed at the OTU level. The size of the nodes indicates the relative abundances of OTUs, and nodes are colored according to phylum (**c**). The red and green lines indicate positive and negative correlations in RAs, respectively. Key topological parameters of bacterial co-occurrence networks are also shown (**d**).

Co-occurrence networks were constructed to visualize the complexity and stability of root-zone soil bacterial community responses to winter cold stress in A201201 and B201810 soils (Fig. 5). The network characteristics varied overall (Fig. 5). The A201201 network consisted of 192 nodes and 228 edges (92 positive and 136 negative edges), an average degree of 2.375, and a modularity of −2.367. The B201810 network comprised 163 nodes and 230 edges (96 positive and 134 negative edges), with an average degree of 2.822 and a modularity of −2.672. The numbers of edges were not significantly different between the A201201 and B201301 networks, but more nodes and higher modularity were observed for A201201 than for B201301. Thus, the A201201 bacterial community structures were more complex and stable than the B201301 bacterial community structures.

### Metabolomics analysis of A201201 and B201810 soils

**Fig 6:**
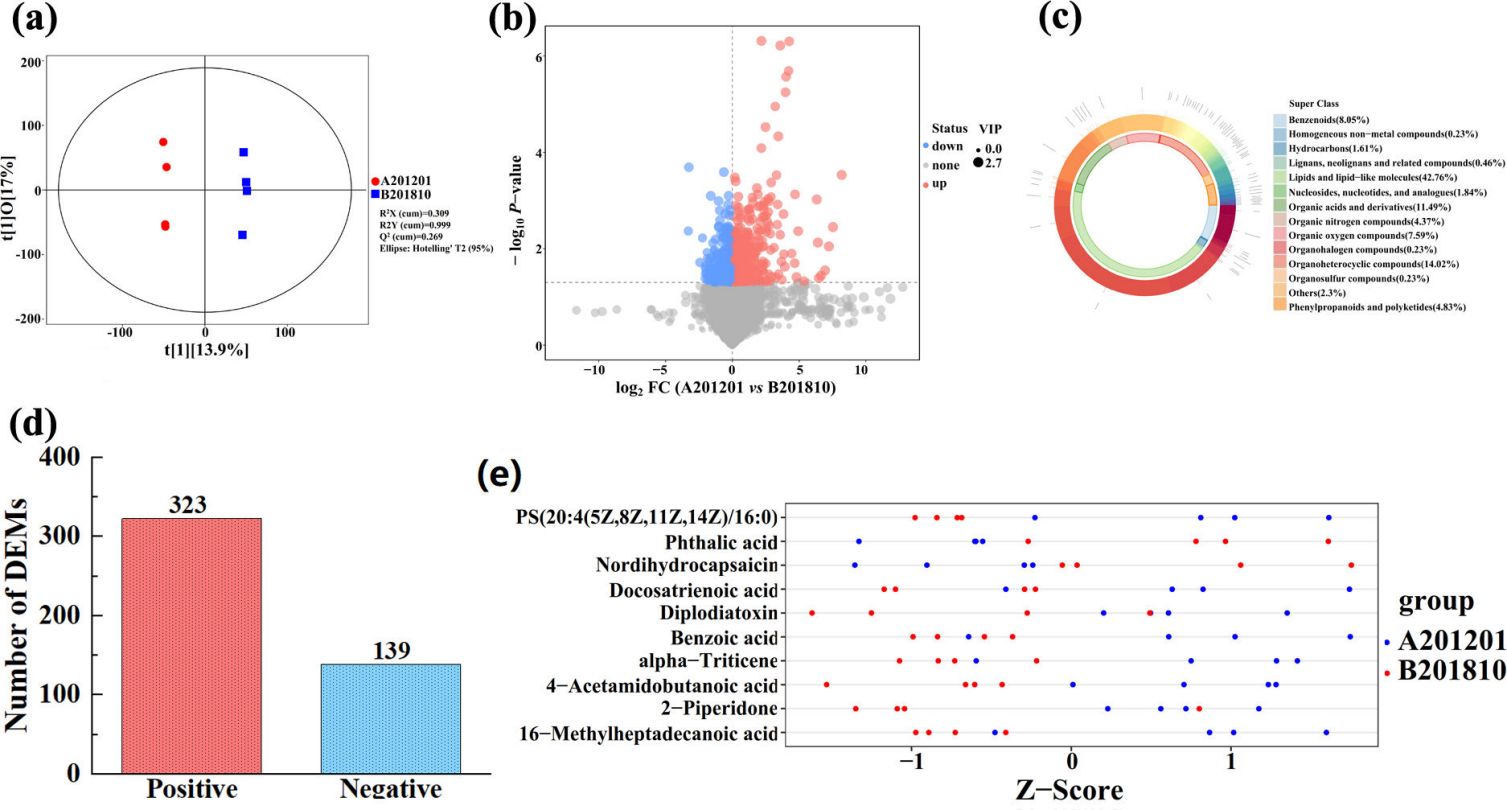
(**a**) Orthogonal projections to latent structures-discriminant analysis score map of A201201 and B201810 root-zone soil metabolomes. A volcano plot showing differentially expressed metabolites (DEMs) between soils A201201 and B201810 (**b**). (**c**) Donut plot showing metabolite classification and proportion. The number of DEMs presents in soils A201201 and B201810 (**d**). *Z*-score plot for soil comparisons between group A201201 and B201810 (**e**).

A total of 19,221 peaks were identified in the metabolomics analysis of root-zone soils of A201201 and B201810, with 462 metabolites identified by ultra-high-performance liquid chromatography coupled to mass spectrometry (UHPLC-MS) analysis. Moreover, orthogonal projections to latent structures-discriminant analysis (OPLS-DA) was used to identify variables that differentiated between A201201 and B201810 soils (Fig. 6a). A201201 and B201810 soils were effectively separated (Fig. 6a), as reflected by the sample distributions of the A201201 and B201810 samples in the positive and negative directions of *t*[1], respectively, with corresponding model values of *R*^2^*X* (cum) = 0.309, *R*^2^*Y* (cum) = 0.999, and *Q*^2^ (cum) = 0.269. Thus, the metabolites of A201201 and B201810 soils significantly differed. This supposition was confirmed by volcano plot which was based on variable importance in projection (VIP) values >1 (*P* < 0.05) (Fig. 6b).

**Fig 7:**
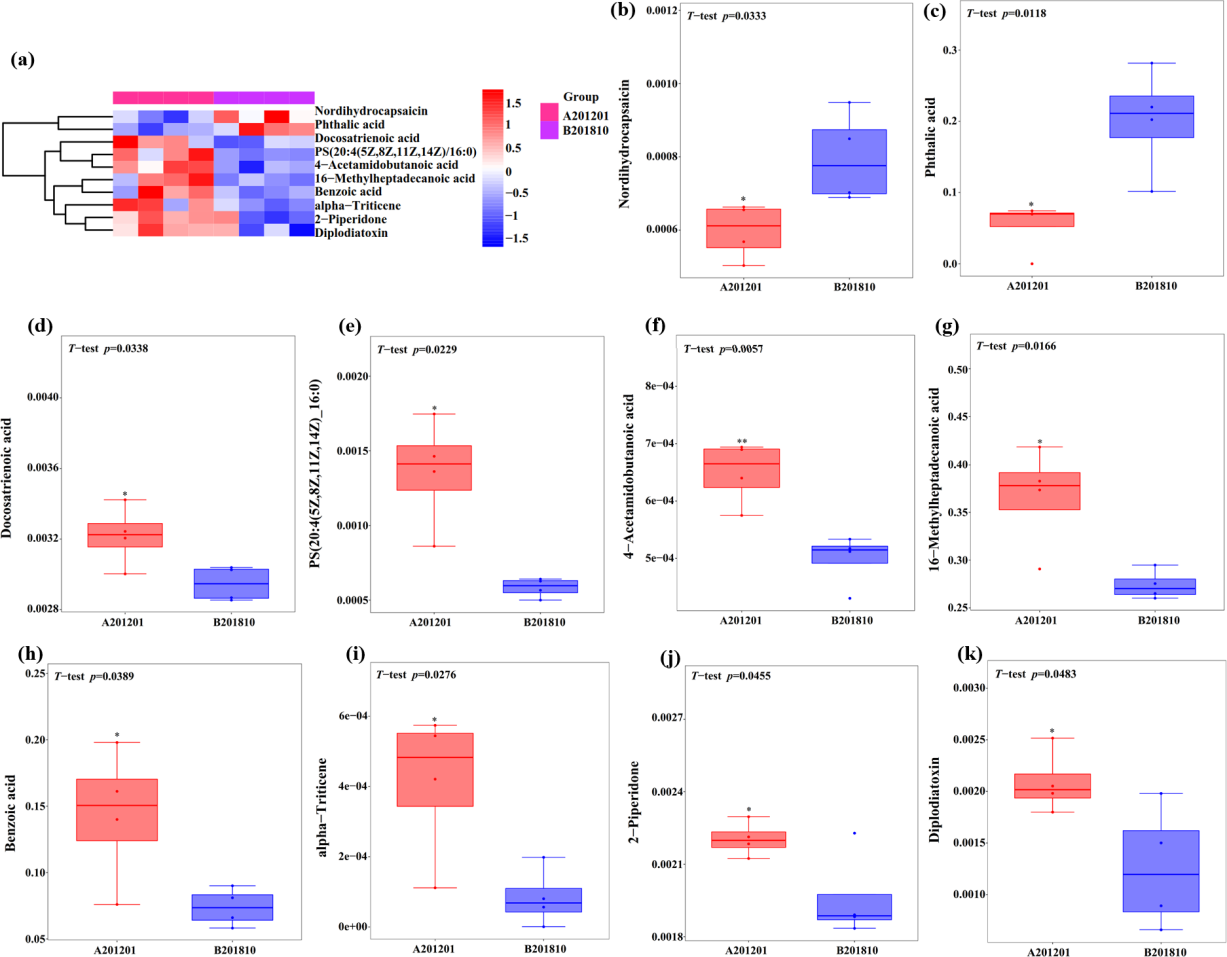
Heatmap showing differential metabolites and metabolome clustering among A201201 and B201810 soils (**a**). Significant DEM abundances between A201201 and B201810 soils (b–k).

The 462 metabolites primarily comprised lipids and lipid-like molecules (42.76%), organoheterocyclic compounds (14.02%), organic acids and derivatives (11.49%), benzenoids (8.05%), and organic oxygen compounds (7.59%) (Fig. 6c), with 323 upregulated and 139 downregulated metabolites (Fig. 6d). After feature scaling via standardization, significant differentially expressed metabolites (DEMs) between A201201 and B201810 were intuitively identified in *Z*-score plots (Fig. 6e). Metabolites with significant differences were also normalized and visualized with a hierarchical clustering heatmap to identify overall metabolite changes (Fig. 7a). Ten DEMs were significantly altered in A201201 soils (including eight DEMs upregulated by 9.2%–391.3% and two DEMs downregulated by 25.1%–73.4%; Table 2; Fig. 7b through k). Specifically, alpha-triticene, PS (20:4(5Z,8Z,11Z,14Z)/16:0), benzoic acid, diplodiatoxin, 16-methylheptadecanoic acid, 4-acetamidobutanoic acid, 2-piperidone, and docosatrienoic acid were upregulated by 391.3%, 132.7%, 94.7%, 65.9%, 33.7%, 30.4%, 12.5%, and 9.2%, respectively, while phthalic acid and nordihydrocapsaicin were downregulated by 73.4% and 25.1%, respectively. Thus, these metabolites may play critical roles in the responses of TDG to winter cold stress.

**TABLE 2:** Differentially expressed metabolites in A201201 and B201810 soils based on variable importance in projection (VIP), fold change (FC), and *P*-values

Compound name	Superclass	VIP	Log2 (FC)	*P*-value	Regulation type
2-Piperidone	–	1.886	0.17	0.046	Up
Diplodiatoxin	Organic acids and derivatives	1.804	0.731	0.048	Up
Benzoic acid	Benzenoids	1.988	0.961	0.039	Up
16-Methylheptadecanoic acid	Lipids and lipid-like molecules	2.187	1.337	0.017	Up
Alpha-triticene	Lipids and lipid-like molecules	1.6	2.297	0.028	Up
4-Acetamidobutanoic acid	Organic acids and derivatives	2.33	1.304	0.006	Up
PS (20:4(5Z,8Z,11Z,14Z)/16:0)	Lipids and lipid-like molecules	2.436	1.219	0.023	Up
Docosatrienoic acid	Lipids and lipid-like molecules	2.015	1.092	0.034	Up
Phthalic acid	Benzenoids	1.401	−1.911	0.012	Down
Nordihydrocapsaicin	Benzenoids	2.003	−0.417	0.033	Down

### Correlation between soil properties, bacterial community composition, and metabolites in A201201 and B201810 soils

**Fig 8:**
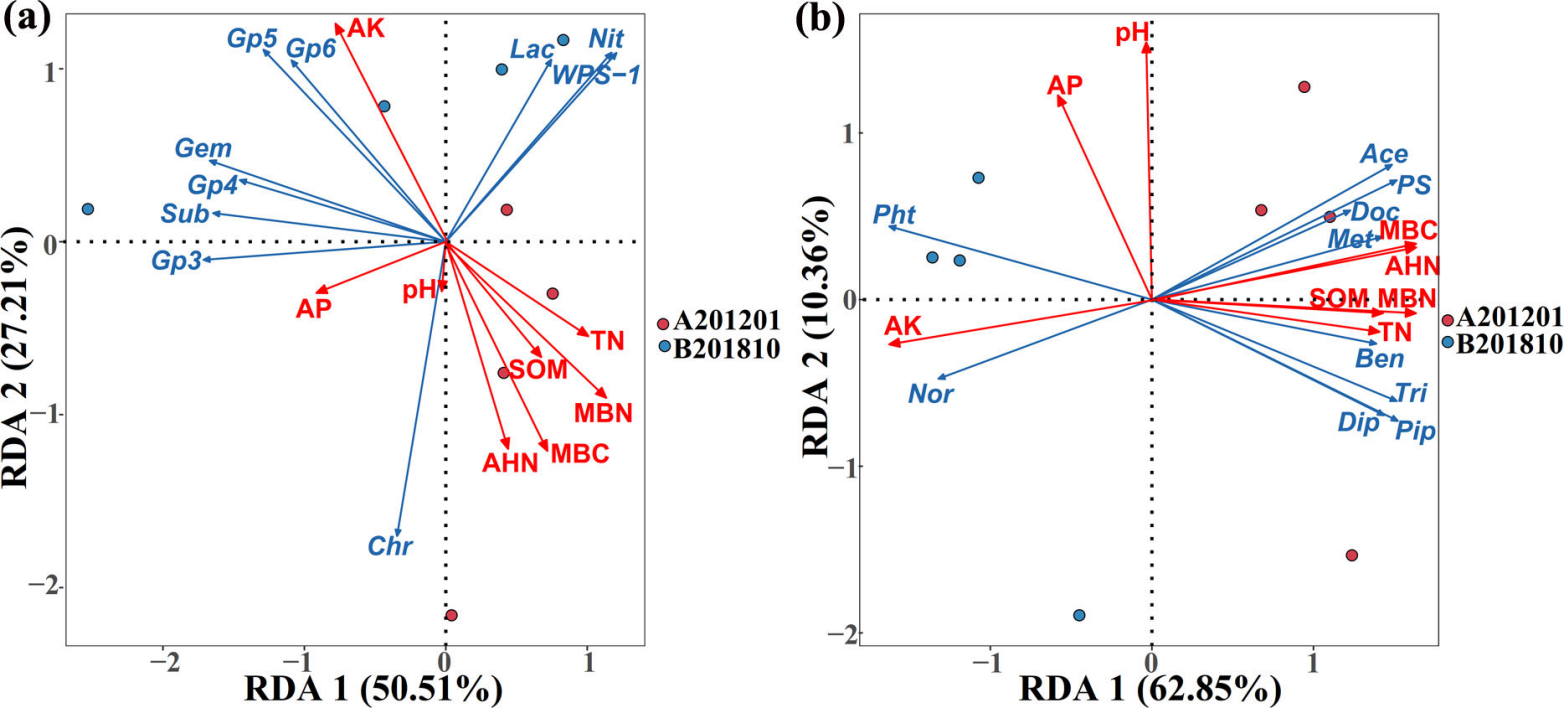
Redundancy discriminant analysis of soil properties is correlated with soil bacterial communities and *Tetrastigma hemsleyanum* Diels & Gilg metabolites. There is a correlation between soil property levels and the relative abundances of the 10 most differential bacterial genera (**a**). Sub, Subdivision3; *Chr*, *Chryseolinea; Lac*, *Lacibacterium*; and *Nit*, *Nitrospira*. Correlations between soil property levels and the 10 significant DEMs in TDG soils (**b**). Pip, 2-piperidone; Dip, diplodiatoxin; Ben, benzoic acid; Met, 16-methylheptadecanoic acid; Tri, alpha-triticene; Ace, 4-acetamidobutanoic acid; PS, PS (20:4(5Z,8Z,11Z,14Z)/16:0); Doc, docosatrienoic acid; Pht, phthalic acid; Nor, nordihydrocapsaicin. Arrows indicate the direction and magnitude of soil properties (pH, SOM, TN, AHN, AP, AK, MBC, and MBN) associated with the different bacterial genera or DEMs.

A201201 soils exhibited significantly higher microbial biomass carbon (MBC) contents and significantly lower available potassium (AK) contents compared to B201810 soils (*P* < 0.05; Table 3), while the levels of soil organic matter (SOM), total nitrogen (TN), alkaline hydrolysis nitrogen (AHN), available phosphorus (AP), microbial biomass nitrogen (MBN), and pH did not clearly differ between the two soils. Redundancy discriminant analysis (RDA) based on genus-level abundances was conducted to determine how soil bacterial community composition correlated with soil environmental factors. At the genus level, a total of 77.72% of the cumulative variance of root-zone bacterial community-factor correction occurred. The seven most important variables (AK, MBN, MBC, AHN, TN, AP, and SOM) explained 73.26%, 66.53%, 62.59%, 50.26%, 40.59%, 28.54%, and 27.79% of the bacterial community variation at the genus level, respectively (Fig. 8a; Table 4). Thus, AK, MBN, MBC, AHN, TN, AP, and SOM were the primary factors associated with bacterial community variation, suggesting that soil nutrient elements clearly influenced bacterial genus-level distributions. To determine the impacts of soil properties on TDG soil metabolites, RDA was also performed to determine correlations between distinct TDG soil metabolites and soil environmental factors (Fig. 8b; Table 4). When considering DEMs, a total of 73.21% of the cumulative variance of root-zone soil metabolite-factor correction was observed. The contribution of the eight main variables (AK, MBC, MBN, AHN, pH, SOM, TN, and AP) explained 96.92%, 93.18%, 88.33%, 83.92%, 70.30%, 61.02%, 59.75%, and 52.81% of soil metabolite variation when considering DEMs, respectively (Fig. 8b; Table 4). Thus, AK, MBC, MBN, AHN, pH, SOM, TN, and AP were the primary factors associated with soil metabolomes, and soil nutrients clearly influenced soil metabolites at the DEM level. Therefore, soil environmental factors appeared to have a significant impact on soil bacterial communities and metabolomes.

**TABLE 3:** Physicochemical properties of A201201 and B201810 soils^*a*,^^*b*^

Parameter	A201201	B201810	Parameter	A201201	B201810
pH	8.09 ± 0.06	8.08 ± 0.05	AP (mg/kg)	29.94 ± 0.83	30.36 ± 2.91
SOM (%)	3.00 ± 0.14	2.79 ± 0.15	AK (mg/kg)	252.67 ± 3.29 #	415.23 ± 5.12
TN (g/kg)	1.94 ± 0.10	1.79 ± 0.12	MBC (mg/kg)	115.37 ± 1.78 *	99.56 ± 1.57
AHN (mg/kg)	81.71 ± 1.23	78.30 ± 1.32	MBN (mg/kg)	36.12 ± 0.97	32.13 ± 0.96

^*a*^ Means are averages ± standard deviations.

^*b*^ * and # indicate statistically significant increases or decreases, respectively, compared to B201810 (*P* < 0.05).

**TABLE 4:** Contribution of soil parameters to variation in bacterial community composition at the genus level or metabolites at the DEM level

Contribution	Soil parameter (%)							
	pH	SOM	TN	AHN	AP	AK	MBC	MBN
**Bacterial genus level** ***(%)***	2.56	27.79	40.59	50.26	28.54	73.26	62.59	66.53
**DEM level** ***(%)***	70.30	61.02	59.75	83.92	52.81	96.92	93.18	88.33

**Fig 9:**
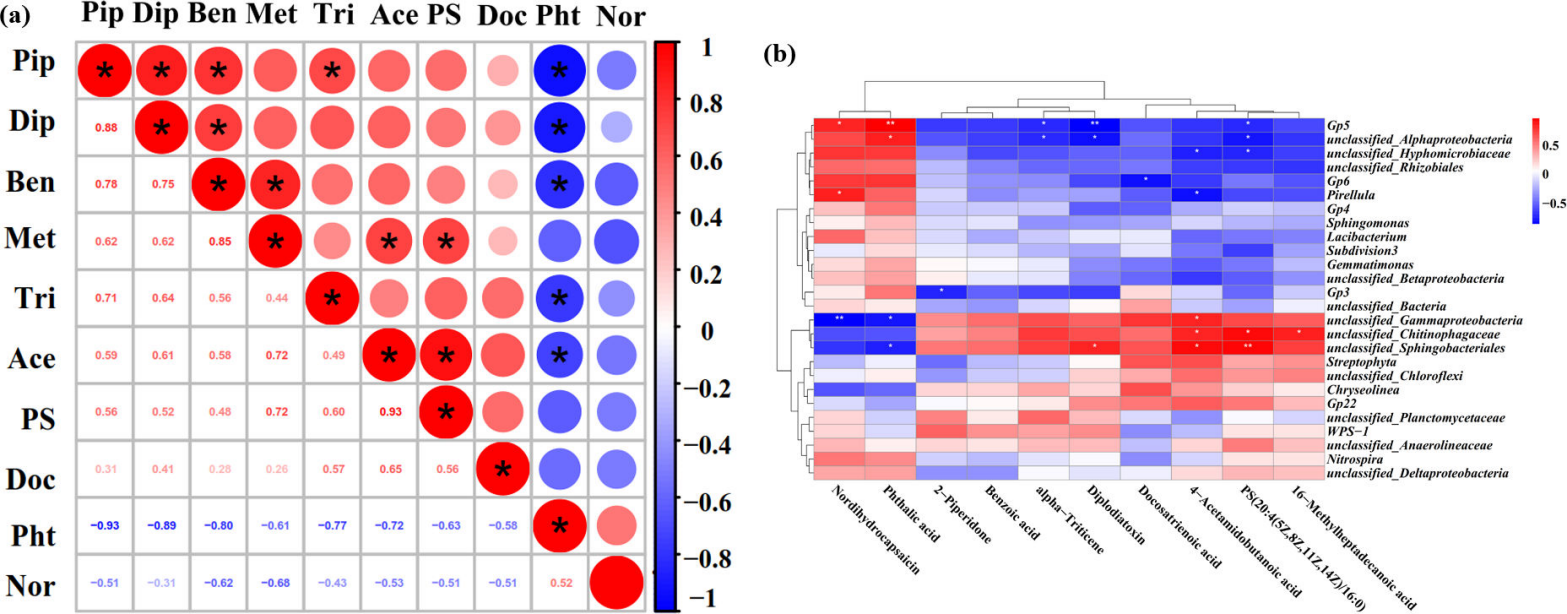
(**a**) Correlation heatmap for differential metabolites between A201201 and B201810 soils. Circle size indicates the degree of correlation between differential metabolites, with red indicating a positive correlation and blue indicating a negative correlation. * indicates a significant correlation. Pip, 2-piperidone; Dip, diplodiatoxin; Ben, benzoic acid; Met, 16-methylheptadecanoic acid; Tri, alpha-triticene; Ace, 4-acetamidobutanoic acid; PS, PS (20:4(5Z,8Z,11Z,14Z)/16:0); Doc, docosatrienoic acid; Pht, phthalic acid; Nor, nordihydrocapsaicin. (**b**) Heatmap of correlations between DEMs and bacterial taxa in A201201 and B201810 soils. * indicates a significant correlation at *P* < 0.05; ** indicates a significant correlation at *P* < 0.01.

A clustergram heatmap was used to further explore relationships between differential metabolites and correlations of differential bacteria with metabolites (Fig. 9). Levels of 2-piperidone were significantly and positively correlated with those of diplodiatoxin, benzoic acid, and alpha-triticene. Diplodiatoxin levels were significantly and positively correlated with those of benzoic acid, while benzoic acid levels were significantly and positively correlated with those of 16-methylheptadecanoic acid. In addition, 16-methylheptadecanoic acid was significantly and positively correlated with 4-acetamidobutanoic acid and PS (20:4(5Z,8Z,11Z,14Z)/16:0), while 4-acetamidobutanoic acid was significantly and positively correlated with PS (20:4(5Z,8Z,11Z,14Z)/16:0). Phthalic acid levels were significantly and negatively correlated with those of diplodiatoxin, benzoic acid, alpha-triticene, and 4-acetamidobutanoic acid (Fig. 9a). Six DEMs between A201201 and B201810 had a significant and positive correlation with six genera of bacteria. These included nordihydrocapsaicin, which was positively correlated with GP5 and *Pirellula* RAs, while phthalic acid was positively correlated with GP5 and unclassified Alphaproteobacteria RAs. Diplodiatoxin was positively correlated with unclassified Sphingobacteriales RAs, while 4-acetamidobutanoic acid was positively correlated with the RAs of unclassified Gammaproteobacteria, unclassified Chitinophagaceae, and unclassified Sphingobacteriales. Furthermore, PS (20:4(5Z,8Z,11Z,14Z)/16:0) was positively correlated with unclassified Chitinophagaceae and unclassified Sphingobacteriales RAs, while 16-methylheptadecanoic acid was positively correlated with unclassified Chitinophagaceae RAs (Fig. 9b). The DEMs in TDG root-zone soils, comprising lipids and lipid-like molecules, in addition to organic acids and derivatives, may play important roles in coordinating root-zone bacterial activities and protecting TDG from cold stress in the winter.

## DISCUSSION

China is the most populous country in the world, and it has been undergoing rapid urbanization, wherein much cultivated land has become occupied by urban expansion (27, 28). Land reclamation is considered a viable option to balance occupation and compensation (29). However, the quality of most reclaimed land cannot generally meet plant requirements for high yields or even successful cultivation due to poor soil fertility and high gravel content (30). TDG is extensively used in Chinese herbal medicine and can grow well in stony soils with few nutrients (13, 14). To increase reclaimed land use, TDG has recently been cultivated in reclaimed lands in Zhejiang Province, China. However, the artificial cultivation of TDG is often limited by winter cold stress due to exposure to 3 months of cold stress every year from December to February. Previous studies have shown that soil microorganisms and metabolites can play essential roles in agriculture ecosystem functioning by influencing soil fertility, crop productivity, and stress tolerance (21, 22, 31). Nevertheless, there is limited information available regarding the systematic investigation of soil bacterial communities, metabolites, and properties related to defense mechanisms in TDG winter cold stress responses within reclaimed lands.

### TDG biomass and quality were different between two varieties

In this study, the cold resistance of A201201 was stronger than that of B201810, while A201201 tuber biomass was significantly higher than that of B201810 after winter cold stress. However, the A201201 TFC was significantly lower than that of B201810. Some previous studies have indicated that the exponential relationships among yields, quality, and resistance to disease are always negative due to functional genomic resource reallocation and genetic linkage drag. For example, dozens of high-yield rice varieties still exist in China but exhibit low quality or susceptibility to blast disease, severely limiting their use in rice production. Breeding for high-quality forage production can reduce seed yield, as evidenced by the resistance of two melon rootstocks to the one and two races of *Fusarium* wilt pathogen, although the quality of grafted scion cultivars was not higher than that of ungrafted controls (32–34).

### Root-zone microbiomes were changed after winter cold stress

Soil microbial diversity is an important indicator for evaluating soil microbial community functioning, particularly as it relates to the ecological significance of microbial communities in maintaining plant health (35, 36). Variation in alpha and beta diversity (based on Chao1, Shannon, Simpson, and Bray-Curtis metrics) was used to investigate changes in bacterial community richness and diversity in the soils of A201201 and B201810. The Simpson index was significantly lower in A201201 soils, although significant differences were not observed in bacterial Chao1 and Shannon index values. Furthermore, PCA revealed some differences in the bacterial community structures of root-zone soils between A201201 and B201810, with variety type accounting for 38.54% of the variation, which is consistent with previous studies. For example, Bogati and Walczak (37) suggested that drought conditions could inhibit the growth of bacteria. Furthermore, Wu et al. (38) observed that microbial communities in the rhizosphere soils of diseased *Panax notoginseng* exhibit decreased alpha diversity relative to healthy plants. Moreover, Ren et al. (35) observed that bacterial and fungal community compositions varied between healthy and diseased bayberry trees. In addition, Wan et al. (39) showed that drought stress reduced the Shannon diversity of soil bacterial communities.

Bacterial communities inhabiting the root zones of A201201 and B201810 soils were investigated using 16S rRNA gene high-throughput sequencing, revealing significant differences in composition. Furthermore, the richness of bacterial OTUs was significantly higher (6.7%) in A201201 soils than in B201810. Proteobacteria, Acidobacteria, and Bacteroidetes were the predominant phyla (RAs >5%) in A201201 and B201810 soils, while the predominant genera (RAs >1%) included Gp6, Gp4, Subdivision3, WPS-1, *Chryseolinea*, Gp3, *Lacibacterium*, *Gemmatimonas*, Gp5, and *Nitrospira.* Proteobacteria, Acidobacteria, and Bacteroidetes are the predominant bacterial taxa in cold winter environments (40–44). The results from this study suggest that additional attention should be given to the roles of *Chryseolinea*, *Lacibacterium*, *Gemmatimonas*, and *Nitrospira* in cold winter environments. *Chryseolinea* have been shown to degrade small organic molecules and large polymers like proteins and polysaccharides, but they might also play important roles in the long-term stability of aerobic granules (45, 46). In addition, *Lacibacterium* can promote pyrene removal in co-contaminated soils (47). *Gemmatimonas*, in addition to improving biofilm formation and denitrification, are also involved in nitrogen fixation and soil organic decomposition (48, 49). *Nitrospira* are key contributors to global nitrogen cycling and also significantly influence nitrification in natural and engineered environments (50, 51). Thus, winter cold stress may reshape root-zone bacterial abundance distributions by altering the abundances of specific soil bacteria associated with TDG in reclaimed lands.

To further elucidate the impacts of winter cold stress on bacterial groups, a LEfSe (LDA >3, *P* < 0.05) analysis was conducted to explore the enrichment of specific bacterial taxa in A201201 and B201810 soils. A total of 35 bacterial biomarker taxa were identified across all soil samples. Furthermore, heatmap visualizations indicated that winter cold stress enriched the abundances of the phyla Bacteroidetes (*P* < 0.05) and Chloroflexi, in addition to the genus *Chryseolinea*, in A201201 soils. Bacteroidetes are well-known degraders of polymeric organic matter and are important components of some organic carbon recycling and decomposition activities (52). Chloroflexi have thick, tough cell walls outside their cell membrane (as gram-positive bacteria), which makes them less susceptible to death under external stress (53). Following winter cold stress, such bacterial cells may have greater potential to colonize and affect soil bacterial communities in reclaimed lands.

Network analysis of taxon co-occurrence patterns provides new insights into the structure of complex microbial communities while complementing and expanding on information provided by alpha and beta diversity metrics (54, 55). Co-occurrence networks were used to analyze the potential interactions between OTUs of A201201 and B201810 soils (SparCC correlation *N* >0.5 or <−0.5, *P* < 0.01). The clustering coefficients can represent the complexity of networks and strong interactions among bacterial taxa (56). The number of network nodes and modularity were higher in A201201 networks compared to B201810 networks, but the average degree decreased. More nodes and edges indicate a more complex network structure (57). Modularity can indicate the integration and connection of ecosystem functional traits (58), and high modularity is also an indicator of a network’s structural stability (59). Thus, a more complex and stable bacterial community structure was observed for A201201 soils compared to B201810 soils.

### Differential root-zone metabolites after winter cold stress

A total of 19,221 metabolomic peaks were identified from all TDG root-zone soil samples. The OPLS-DA of the metabolite profiles revealed that the metabolite compositions were significantly different in A201201 and B201810 soils, as also demonstrated by volcano and *Z*-score plots. A total of 462 metabolites were identified by UHPLC-MS analysis that primarily comprised lipids and lipid-like molecules, organoheterocyclic compounds, organic acids and derivatives, benzenoids, and organic oxygen compounds, with 323 of the metabolites upregulated and 139 downregulated. A total of 10 DEMs were significantly altered in A201201 relative to B201810, among which8 were significantly upregulated [alpha-triticene, PS (20:4(5Z,8Z,11Z,14Z)/16:0), benzoic acid, diplodiatoxin, 16-methylheptadecanoic acid, 4-acetamidobutanoic acid, 2-piperidone, and docosatrienoic acid], and 2 were significantly downregulated (phthalic acid and nordihydrocapsaicin). These differential metabolites comprised a variety of bioactive components such as lipids and lipid-like molecules, organic acids and derivatives, benzenoids, and other secondary metabolites that could be involved in different bioactivities and potentially multiple biochemical processes related to TDG growth. Bioactive compounds have previously been shown to be products of long-term adaptation to specific environments for medicinal plants that comprise secondary metabolites that accumulate in response to abiotic and biotic stresses (60, 61). Mahajan et al. (62) showed that many medicinal and aromatic plants accumulate secondary metabolites (such as terpenoids, phenolics, and nitrogen-containing compounds) in response to various kinds of abiotic stresses (such as drought, temperature, salts, and heavy metals) for survival, and these compounds act as an interface with the adverse environment (62). Moreover, secondary metabolites (such as terpenes, terpenoids, alkaloids, and phenolics) can aid plants in building metabolism for disease resistance, even developing complex defense systems against various invading pathogens (63).

### Correlation analysis between soil properties, microbiomes, and metabolites

External stresses can affect soil bacterial community structures through various environmental factors, such as soil fertility, temperature, and rainfall (64). RDA was used to examine the correlations between bacterial community components and environmental factors and assess whether soil physicochemical properties influenced bacterial composition dispersion after winter cold stress. A total of 77.72% of the cumulative variance of root-zone bacterial community-factor correction was explained at the genus level in the RDA. Furthermore, bacterial communities were significantly influenced by AK, MBN, MBC, AHN, TN, AP, and OMC, which explained 73.26%, 66.53%, 62.59%, 50.26%, 40.59%, 28.54%, and 27.79% of bacterial community variation, respectively. The growth of soil bacteria is affected by diverse environmental factors. For example, Wan et al. (39) observed that soil moisture content was the main factor affecting soil microbial community structure. Li et al. (12) also reported that the growth of pakchoi soil bacteria was affected by AP, AHN, pH, OMC, and TN. Furthermore, Li et al. (29) observed that AK, OMC, AP, and MBN were the main factors affecting the bacterial community structures of corn in reclaimed barren soils.

RDA was also used in this study to examine the correlations between soil metabolites and environmental factors. A total of 73.21% of the cumulative variance in root-zone soil metabolite-factor corrections was explained at the DEM level, and soil metabolites were significantly influenced by AK, MBC, MBN, AHN, pH, SOM, TN, and AP that explained 96.92%, 93.18%, 88.33%, 83.92%, 70.30%, 61.02%, 59.75%, and 52.81% of the soil metabolite compositions in A201201 and B201810, respectively. Tang et al. (65) reported that adenine and adenosine levels were significantly and positively correlated with total P, total K, and pH in a sugarcane/peanut intercropping system. Furthermore, N has been shown to significantly affect plant metabolomes, which could then impact amino acids, carbohydrates, and secondary metabolites (66, 67).

To further investigate the correlation between differential metabolites, a heatmap of metabolite correlations was evaluated. The relationships among differential metabolites were complicated and comprised some positive and negative correlations. A clustergram heatmap was generated to explore the correlations between bacterial taxa and DEMs. Nine DEMs were significantly correlated with nine different bacterial genera, with Gp5 RAs significantly and positively correlated with two DEMs but negatively correlated with three DEMs. Furthermore, unclassified Alphaproteobacteria RAs were significantly and positively correlated with one DEM but negatively correlated with three DEMs, while unclassified Hyphomicrobiaceae RAs were significantly and negatively correlated with two DEMs. Furthermore, GP6 and GP3 RAs were significantly and negatively correlated with one DEM, while *Pirellula* RAs were significantly and positively correlated with one DEM, as well as negatively correlated with one DEM. The RAs of unclassified Gammaproteobacteria were significantly and negatively correlated with two DEMs, as well as positively correlated with one DEM. Finally, unclassified Chitinophagaceae RAs were significantly and positively correlated with three DEMs, while unclassified Sphingobacteriales RAs were significantly and negatively correlated with one DEM but positively correlated with three DEMs. Thus, complicated interactions were suggested between bacterial taxa and DEMs within TDG root-zone soils. As we know, soil metabolites could reflect the biological responses of soil microbes to different conditions (68). For instance, under plastic greenhouse cultivation, 11 DEMs positively or negatively correlated with the RAs of differential bacteria were detected between the pepper rhizosphere and bulk soils (69). Bi et al. (70) showed that some metabolites (such as diterpenoids and fatty acids) were significantly negatively correlated with some microbes (like *Rubrobacteria*, *Ascomycota*, and *Glomeromycota*) under drought stress.

## MATERIALS AND METHODS

### Plant materials and field experimental design

The field experiment was completely randomly designed and conducted between 4 August 2019 and 14 February 2023. TDG cutting seedlings were planted in reclaimed land within Shilinhou Village in Chun’an country, Zhejiang Province, China. The area of each plot was 12 m^2^ (length: 24 m, width: 0.5 m, height: 30 cm), and adjacent ridges were spaced 30 cm apart. The cutting seedlings were planted on the ridges in a single row with a 30-cm space between them. The cold-tolerant variety (A201201) and the cold-intolerant variety (B201810) were used for the study and were obtained from the Hangzhou Academy of Agricultural Sciences (Hangzhou, China). On 4 August 2019, the top 0–20 cm of soil in the experimental fields was mixed with sheep manure (2.25 kg/m^2^) and plant ash (0.15 kg/m^2^) before planting. The cutting seedlings of A201201 and B201810 were then planted in the reclaimed land, with three replicates for each treatment.

### Measurement of tuber parameters and soil properties

Tuber samples were collected for various measurements. On 14 February 2023 (about 42 months after planting), three plants were randomly selected from each plot, and all tubers from each plant were harvested from the soil using hoes. After reaping, tuber fresh weights were immediately measured using an electronic balance (Shanghai Precision Instrument Co., Ltd., Shanghai, China) after removing soil with tap water. The lengths and widths of tubers were measured using a digital caliper (Ningbo Great Wall Precision Industrial Co., Ltd., Yuyao, China). Tuber dry weights were measured after air drying for 3 months. The TFC of dry tubers was determined using the NaNO_2_-Al(NO_3_)_3_-NaOH colorimetric method (71).

In addition, soil properties were evaluated, including pH, SOM, TN, AHN, AP, AK, MBC, and MBN, as previously described (72–80). Upon tuber harvesting, about 1.0 kg of fresh root-zone soil (5–20 cm) was simultaneously sampled from each plant, with three replicate plants sampled from each plot. After air drying at room temperature and passing through 0.45 mm sieves to remove stones and root debris, soil properties were measured. Soil pH was measured using a pH meter (FE28, MettlerToledo, Zurich, Switzerland) and a soil-to-water ratio of 1:5 (g/mL). SOM was measured using K_2_Cr_2_O_7_ extraction oxidation with external heating, while TN was determined spectrophotometrically. AHN was quantified by conductometric titration, while AP and AK were extracted with an ammonium lactate solution and detected with spectrophotometry and flame photometry. MBC and MBN were determined after chloroform fumigation extraction. All treatments consisted of three replicates.

### Soil microbial community sequencing

During tuber harvesting, 10 g of root-zone soil from each plant, with three replicate plants from each plot, was sampled and stored at −80°C for subsequent molecular analyses. Soil genomic DNA was extracted from each sample using a DNA extraction kit (E.Z.N.A Mag-Bind Soil DNA Kit, OMEGA, Norcross, GA, USA) according to the manufacturer’s instructions. The quality of the extracted DNA was analyzed using a NanoDrop 1000 spectrophotometer (Thermo Fisher Scientific, MA, USA) (81). The universal PCR primer sets 341F (5′–CCTACGGGNGGCWGCAG–3′) and 805R (5′–GACTACHVGGGTATCTAATCC–3′) (82) were used to amplify bacterial 16S rRNA genes from the V3-V4 hypervariable regions. After PCR amplification, amplicons were purified using Vazyme VAHTSTM DNA clean beads (Vazyme, Nanjing, China) and quantified using the Qubit dsDNA HS Assay Kit (Thermo Fisher, MA, USA). The samples were pooled in equal concentrations and sequenced using paired-end (2 × 250 bp) sequence chemistry on the Illumina MiSeq/Hiseq platform (Shanghai Sangon Biotechnology Co., Ltd., Shanghai, China).

Bioinformatics analysis of bacterial sequencing data was conducted as previously described (12). Briefly, raw reads were quality filtered using PEAR (v.0.9.8) to merge paired-end reads (83) and PRINSEQ to remove low-quality reads (average quality score <20) (84). After primers were trimmed using Cutadapt (v.1.18) (85), clean reads were clustered into OTUs at the 97% nucleotide similarity level with Usearch (86, 87). Representative reads for each OTU were chosen using QIIME (v.2020.06) (88), followed by taxonomic annotation of representative 16S rRNA genes and BLAST searches against the RDP database using the RDP classifier and a confidence threshold of 90% (89).

### LC-MS metabolomics assays

During tuber harvesting, 10 g of root-zone soil from each plant, including four plants from each plot, was sampled and stored at −80°C for subsequent metabolomic analysis. Liquid chromatography mass spectrometry analyses were conducted using a UHPLC system (Vanquish, Thermo Fisher Scientific) with a UPLC HSS T3 column (2.1 × 100 mm, 1.8 µm) coupled to an Orbitrap Exploris 120 mass spectrometer (Orbitrap MS, Thermo), as previously described (29). To ensure repeatability and reliability of the analyses, a quality control sample (generally prepared by mixing an equal aliquot of supernatants from all samples) was included in the analytical measurements. Raw data were converted to the mzXML format using ProteoWizard, processed with an in-house program, and then analyzed in R based on XCMS. An in-house MS2 database (Sangon) was then used for metabolite annotation, with the cutoff set at 0.3.

### Statistical analyses

Preliminary data analysis was conducted in Excel 2007. One-way ANOVA tests were performed using the SPSS software program (v.16.0) (SPSS Inc., Chicago, IL, USA). Various diversity indices, including richness estimation (Chao1) and alpha diversity indices (i.e., Shannon and Simpson indices), were calculated using the Origin software program (v.2023) (Hampton, MA, USA). Beta diversity analysis was conducted by PCA based on Bray-Curtis distances of community compositional variation (90). LEfSe was used to identify biomarker taxa for the A201201 and B201810 communities, in addition to estimating biomarker effect sizes (91). RDA was used to identify the major environmental variables associated with bacterial communities and soil metabolites (92, 93). Co-occurrence network analysis was conducted using a Spearman correlation matrix, based on high RAs (>1%) and statistically significant correlations of RAs (*P* < 0.01, Spearman’s coefficient *N* >0.5 or <−0.5) among OTUs (94). OPLS-DA, volcano plot visualizations, and heatmap visualizations were generated using the MetaboAnalyst 4.0 platform to investigate metabolite levels for each group. A differential metabolite correlation heatmap was visualized using Pearson’s correlation coefficients between all metabolites (95). A cluster heatmap was also used to investigate the correlations between DEMs (considering those with the largest VIP values and *P* < 0.05) and the 26 bacterial genera (96).

### Conclusions

In conclusion, significant differences were observed in soil bacterial communities, metabolites, and properties between the cold-tolerant TDG variety (A201201) and the cold-intolerant variety (B201810) following winter cold stress. Specifically, the OTUs number was significantly higher, and a more complex and stable bacterial community structure was observed in A201201 soils than that of B201810. A total of 35 bacterial biomarker taxa were identified across all A201201 and B201810 soils. The abundances of the phyla Bacteroidetes, Chloroflexi, and the genus *Chryseolinea* were significantly enriched in A201201 soils. A total of 462 metabolites were identified in all A201201 and B201810 soils, among which 10 DEMs (belonging to lipids and lipid-like molecules, organic acids and derivatives, and benzenoids, significantly correlated with nine bacterial genera) were significantly altered in A201201 relative to B201810. In addition, RDA indicated that soil physiochemical properties were related to variations in bacterial community composition and metabolite profiles. Overall, more richness, complex and stable bacterial community structure, specific bacteria and metabolites, and higher AK, MBC, MBN, AHN, SOM, TN, and AP may protect TDG against winter cold stress in reclaimed land. As a result, this study provides new insights into the TDG defense mechanisms involved in winter cold tress responses when grown on reclaimed land, as well as practical guidelines for achieving optimal TDG production.

## Data Availability

The data presented in this study can be found in online repositories under the accession numbers PRJNA1077642 (NCBI) and CNP0004457 (CNGBdb), respectively.

## References

[1] Zhu R, Xu X, Ying J, Cao G, Wu X. 2020. The phytochemistry, pharmacology, and quality control of Tetrastigma hemsleyanum Diels & Gilg in China: a review. Front Pharmacol 11:550497. doi:10.3389/fphar.2020.550497 33101019 PMC7546407

[2] Xiang Q, Hu S, Ligaba-Osena A, Yang J, Tong F, Guo W. 2021. Seasonal variation in transcriptomic profiling of Tetrastigma hemsleyanum fully developed tuberous roots enriches candidate genes in essential metabolic pathways and phytohormone signaling. Front Plant Sci 12:659645. doi:10.3389/fpls.2021.659645 34305963 PMC8300961

[3] Wang Y-H, Jiang W-M, Comes HP, Hu FS, Qiu Y-X, Fu C-X. 2015. Molecular phylogeography and ecological niche modelling of a widespread herbaceous climber, Tetrastigma hemsleyanum (Vitaceae): insights into Plio-Pleistocene range dynamics of evergreen forest in subtropical China. New Phytol 206:852–867. doi:10.1111/nph.13261 25639152

[4] Peng X, Wu H, Chen H, Zhang Y, Qiu D, Zhang Z. 2019. Transcriptome profiling reveals candidate flavonol-related genes of Tetrastigma hemsleyanum under cold stress. BMC Genomics 20:687. doi:10.1186/s12864-019-6045-y 31472675 PMC6717372

[5] Hu W, Zheng Y, Xia P, Liang Z. 2021. The research progresses and future prospects of Tetrastigma hemsleyanum Diels et Gilg: a valuable Chinese herbal medicine. J Ethnopharmacol 271:113836. doi:10.1016/j.jep.2021.113836 33465440

[6] Ji T, Ji WW, Wang J, Chen HJ, Peng X, Cheng KJ, Qiu D, Yang WJ. 2021. A comprehensive review on traditional uses, chemical compositions, pharmacology properties and toxicology of Tetrastigma hemsleyanum. J Ethnopharmacol 264:113247. doi:10.1016/j.jep.2020.113247 32800929 PMC7422820

[7] Ji Q, Cheng W, Wu H. 2014. Study on biological characteristics of Radix Tetrastigmue. Shizhen Guoyi Guoyao 25:219–221. doi:10.3969/j.issn.1008-0805.2014.01.095

[8] Peng X, Ji Q, Liang Y, Zhang Y, Lou T. 2016. Research progress in utilization of Tetrastigma hemsleyanum germplasms. Zhongguo Xiandai Zhongyao 18:1088–1092. doi:10.13313/j.issn.1673-4890.2016.8.034

[9] Hu W, Xia P, Liang Z. 2021. Molecular cloning and structural analysis of key enzymes in Tetrastigma hemsleyanum for resveratrol biosynthesis. Int J Biol Macromol 190:19–32. doi:10.1016/j.ijbiomac.2021.08.178 34478792

[10] Peng X, Ji Q, Fan S, Zhang Y, Zhang J. 2015. Genetic diversity in populations of the endangered medicinal plant Tetrastigma hemsleyanum revealed by ISSR and SRAP markers: implications for conservation. Genet Resour Crop Evol 62:1069–1078. doi:10.1007/s10722-014-0210-6

[11] Liu Y, Pan J, Ni S, Xing B, Cheng K, Peng X. 2022. Transcriptome and metabonomics combined analysis revealed the defense mechanism involved in hydrogen-rich water-regulated cold stress response of Tetrastigma hemsleyanum. Front Plant Sci 13:889726. doi:10.3389/fpls.2022.889726 35812920 PMC9260428

[12] Li X, Li D, Jiang Y, Xu J, Ren X, Zhang Y, Wang H, Lu Q, Yan J, Ahmed T, Li B, Guo K. 2023. The effects of microbial fertilizer based Aspergillus brunneoviolaceus HZ23 on pakchoi growth, soil properties, rhizosphere bacterial community structure, and metabolites in newly reclaimed land. Front Microbiol 14:1091380. doi:10.3389/fmicb.2023.1091380 36814570 PMC9939755

[13] Hong C, Shao Q, Qin W, Zhang J, Wei B, Shen D, Zheng B, Guo H. 2021. Bacterial communities are associated with the tuber size of Tetrastigma hemsleyanum in stony soils. Biol Fertil Soils 57:373–388. doi:10.1007/s00374-020-01530-4

[14] Fu L-Z, Zhao L-M, Lyu H-Q, Yan M-Q, Zheng Y-Q, Liu Q, Jin L, Cheng J-W, Lu T-G, Wang L-Y. 2019. Effects of nitrogen level on growth of Tetrastigma hemsleyanum and phytochemical content and antioxidant activity in stems and leaves. Zhongguo Zhong Yao Za Zhi 44:696–702. doi:10.19540/j.cnki.cjcmm.20181204.006 30989881

[15] Einset J, Winge P, Bones A. 2007. ROS signaling pathways in chilling stress. Plant Signal Behav 2:365–367. doi:10.4161/psb.2.5.4461 19704600 PMC2634213

[16] Soualiou S, Duan FY, Li X, Zhou WB. 2022. Crop production under cold stress: an understanding of plant responses, acclimation processes, and management strategies. Plant Physiol Biochem 190:47–61. doi:10.1016/j.plaphy.2022.08.024 36099808

[17] Hussain S, Khaliq A, Ali B, Hussain HA, Qadir T, Hussain S. 2018. Temperature extremes: impact on rice growth and development, p 153–171. In Plant abiotic stress tolerance. Springer, Cham.

[18] Bhattacharya A. 2022. Effect of low temperature stress on photosynthesis and allied traits: a review, p 199–297. In Physiological processes in plants under low temperature stress. Springer, Singapore.

[19] Arshad MS, Farooq M, Asch F, Krishna JSV, Prasad PVV, Siddique KHM. 2017. Thermal stress impacts reproductive development and grain yield in rice. Plant Physiol Biochem 115:57–72. doi:10.1016/j.plaphy.2017.03.011 28324683

[20] Waadt R, Seller CA, Hsu PK, Takahashi Y, Munemasa S, Schroeder JI. 2022. Plant hormone regulation of abiotic stress responses. Nat Rev Mol Cell Biol 23:680–694. doi:10.1038/s41580-022-00479-6 35513717 PMC9592120

[21] Abbasi AO, Salazar A, Oh Y, Reinsch S, del Rosario Uribe M, Li J, Rashid I, Dukes JS. 2020. Reviews and syntheses: soil responses to manipulated precipitation changes-an assessment of meta-analyses. Biogeosciences 17:3859–3873. doi:10.5194/bg-17-3859-2020

[22] Barria C, Malecki M, Arraiano CM. 2013. Bacterial adaptation to cold. Microbiology (Reading) 159:2437–2443. doi:10.1099/mic.0.052209-0 24068238

[23] Zhang Y, Tao Y, Zhang H, Wang L, Sun G, Sun X, Erinle KO, Feng C, Song Q, Li M. 2015. Effect of di-n-butyl phthalate on root physiology and rhizosphere microbial community of cucumber seedlings. J Hazard Mater 289:9–17. doi:10.1016/j.jhazmat.2015.01.071 25702635

[24] Zhao L, Zhang H, White JC, Chen X, Li H, Qu X, Ji R. 2019. Metabolomics reveals that engineered nanomaterial exposure in soil alters both soil rhizosphere metabolite profiles and maize metabolic pathways. Environ Sci Nano 6:1716–1727. doi:10.1039/C9EN00137A

[25] Chen D, Hou Q, Jia L, Sun K. 2021. Combined use of two Trichoderma strains to promote growth of pakchoi (Brassica chinensis L.). Agronomy 11:726. doi:10.3390/agronomy11040726

[26] Ren H, Wang H, Yu Z, Zhang S, Qi X, Sun L, Wang Z, Zhang M, Ahmed T, Li B. 2021. Effect of two kinds of fertilizers on growth and rhizosphere soil properties of bayberry with decline disease. Plants (Basel) 10:2386. doi:10.3390/plants10112386 34834750 PMC8624721

[27] Tan M, Li X, Lu C. 2005. Urban land expansion and arable land loss of the major cities in China in the 1990s. Sci China Ser D Earth Sci 48:1492–1500. doi:10.1360/03yd0374

[28] Wang S, Bai X, Zhang X, Reis S, Chen D, Xu J, Gu B. 2021. Urbanization can benefit agricultural production with large-scale farming in China. Nat Food 2:183–191. doi:10.1038/s43016-021-00228-6 37117442

[29] Li X, Wang D, Lu Q, Tian Z, Yan J. 2023. Effects of SMOF on soil properties, root-zone microbial community structure, metabolites, and maize (Zea mays L.) response on a reclaimed barren mountainous land. Front Microbiol 14:1181245. doi:10.3389/fmicb.2023.1181245 37303787 PMC10248427

[30] Li X, Su Y, Ahmed T, Ren H, Javed MR, Yao Y, An Q, Yan J, Li B. 2021. Effects of different organic fertilizers on improving soil from newly reclaimed land to crop soil. Agriculture 11:560. doi:10.3390/agriculture11060560

[31] Hartmann M, Six J. 2023. Soil structure and microbiome functions in agroecosystems. Nat Rev Earth Environ 4:4–18. doi:10.1038/s43017-022-00366-w

[32] Trionfetti Nisini P, Colla G, Granati E, Temperini O, Crinò P, Saccardo F. 2002. Rootstock resistance to fusarium wilt and effect on fruit yield and quality of two muskmelon cultivars. Sci Hortic 93:281–288. doi:10.1016/S0304-4238(01)00335-1

[33] Xiao N, Pan C, Li Y, Wu Y, Cai Y, Lu Y, Wang R, Yu L, Shi W, Kang H, Zhu Z, Huang N, Zhang X, Chen Z, Liu J, Yang Z, Ning Y, Li A. 2021. Genomic insight into balancing high yield, good quality, and blast resistance of japonica rice. Genome Biol 22:283. doi:10.1186/s13059-021-02488-8 34615543 PMC8493723

[34] Jaškūnė K, Kemešytė V, Aleliūnas A, Statkevičiūtė G. 2022. Genome-wide markers for seed yield and disease resistance in perennial ryegrass. Crop J 10:508–514. doi:10.1016/j.cj.2021.07.005

[35] Ren H, Wang H, Qi X, Yu Z, Zheng X, Zhang S, Wang Z, Zhang M, Ahmed T, Li B. 2021. The damage caused by decline disease in bayberry plants through changes in soil properties, rhizosphere microbial community structure and metabolites. Plants (Basel) 10:2083. doi:10.3390/plants10102083 34685892 PMC8540645

[36] Zhang Y, Dong S, Gao Q, Liu S, Zhou H, Ganjurjav H, Wang X. 2016. Climate change and human activities altered the diversity and composition of soil microbial community in alpine grasslands of the Qinghai-Tibetan plateau. Sci Total Environ 562:353–363. doi:10.1016/j.scitotenv.2016.03.221 27100015

[37] Bogati K, Walczak M. 2022. The impact of drought stress on soil microbial community, enzyme activities and plants. Agronomy 12:189. doi:10.3390/agronomy12010189

[38] Wu Z, Hao Z, Zeng Y, Guo L, Huang L, Chen B. 2015. Molecular characterization of microbial communities in the rhizosphere soils and roots of diseased and healthy Panax notoginseng. Antonie Van Leeuwenhoek 108:1059–1074. doi:10.1007/s10482-015-0560-x 26296378

[39] Wan Q, Li L, Liu B, Zhang Z, Liu Y, Xie M. 2023. Different and unified responses of soil bacterial and fungal community composition and predicted functional potential to 3 years’ drought stress in a semiarid alpine grassland. Front Microbiol 14:1104944. doi:10.3389/fmicb.2023.1104944 37082184 PMC10112540

[40] Liu Y, Priscu JC, Yao T, Vick-Majors TJ, Xu B, Jiao N, Santibáñez P, Huang S, Wang N, Greenwood M, Michaud AB, Kang S, Wang J, Gao Q, Yang Y. 2016. Bacterial responses to environmental change on the Tibetan Plateau over the past half century. Environ Microbiol 18:1930–1941. doi:10.1111/1462-2920.13115 26530871

[41] Zeng Y-X, Yu Y, Liu Y, Li H-R. 2016. Psychrobacter glaciei sp. nov., isolated from the ice core of an Arctic glacier. Int J Syst Evol Microbiol 66:1792–1798. doi:10.1099/ijsem.0.000939 26827927

[42] Shen L, Liu Y, Wang N, Jiao N, Xu B, Liu X. 2018. Variation with depth of the abundance, diversity and pigmentation of culturable bacteria in a deep ice core from the Yuzhufeng Glacier, Tibetan Plateau. Extremophiles 22:29–38. doi:10.1007/s00792-017-0973-8 29071425

[43] Belov AA, Cheptsov VS, Manucharova NA, Ezhelev ZS. 2020. Bacterial communities of Novaya Zemlya archipelago ice and permafrost. Geosciences 10:67. doi:10.3390/geosciences10020067

[44] Paun VI, Lavin P, Chifiriuc MC, Purcarea C. 2021. First report on antibiotic resistance and antimicrobial activity of bacterial isolates from 13,000-year old cave ice core. Sci Rep 11:514. doi:10.1038/s41598-020-79754-5 33436712 PMC7804186

[45] Xu J, He J, Wang M, Li L. 2018. Cultivation and stable operation of aerobic granular sludge at low temperature by sieving out the batt-like sludge. Chemosphere 211:1219–1227. doi:10.1016/j.chemosphere.2018.08.018 30223338

[46] Zhang H, Liu Y-Q, Mao S, Steinberg CEW, Duan W, Chen F. 2022. Reproducibility of aerobic granules in treating low-strength and low-C/N-ratio wastewater and associated microbial community structure. Processes 10:444. doi:10.3390/pr10030444

[47] Li G, Wang Z, Lv Y, Jia S, Chen F, Liu Y, Huang L. 2021. Effect of culturing ryegrass (Lolium perenne L.) on CD and pyrene removal and bacteria variations in co-contaminated soil. Environ Technol Innov 24:101963. doi:10.1016/j.eti.2021.101963

[48] Lupwayi NZ, Larney FJ, Blackshaw RE, Kanashiro DA, Pearson DC, Petri RM. 2017. Pyrosequencing reveals profiles of soil bacterial communities after 12 years of conservation management on irrigated crop rotations. Appl Soil Ecol 121:65–73. doi:10.1016/j.apsoil.2017.09.031

[49] Peng C, Gao Y, Fan X, Peng P, Huang H, Zhang X, Ren H. 2019. Enhanced biofilm formation and denitrification in biofilters for advanced nitrogen removal by rhamnolipid addition. Bioresour Technol 287:121387. doi:10.1016/j.biortech.2019.121387 31076293

[50] Daebeler A, Kitzinger K, Koch H, Herbold CW, Steinfeder M, Schwarz J, Zechmeister T, Karst SM, Albertsen M, Nielsen PH, Wagner M, Daims H. 2020. Exploring the upper pH limits of nitrite oxidation: diversity, ecophysiology, and adaptive traits of haloalkalitolerant Nitrospira. ISME J 14:2967–2979. doi:10.1038/s41396-020-0724-1 32709974 PMC7784846

[51] Latocheski EC, da Rocha MCV, Braga MCB. 2022. Nitrospira in wastewater treatment: applications, opportunities and research gaps. Rev Environ Sci Biotechnol 21:905–930. doi:10.1007/s11157-022-09634-z

[52] Thomas F, Hehemann JH, Rebuffet E, Czjzek M, Michel G. 2011. Environmental and gut bacteroidetes: the food connection. Front Microbiol 2:93. doi:10.3389/fmicb.2011.00093 21747801 PMC3129010

[53] Manzoni S, Schimel JP, Porporato A. 2012. Responses of soil microbial communities to water stress: results from a meta-analysis. Ecology 93:930–938. doi:10.1890/11-0026.1 22690643

[54] Barberán A, Bates ST, Casamayor EO, Fierer N. 2012. Using network analysis to explore co-occurrence patterns in soil microbial communities. ISME J 6:343–351. doi:10.1038/ismej.2011.119 21900968 PMC3260507

[55] Jiang H, Lv L, Ahmed T, Jin S, Shahid M, Noman M, Osman H-EH, Wang Y, Sun G, Li X, Li B. 2022. Effect of the nanoparticle exposures on the tomato bacterial wilt disease control by modulating the rhizosphere bacterial community. Int J Mol Sci 23:414. doi:10.3390/ijms23010414 PMC8745216 35008839

[56] Guo B, Zhang L, Sun H, Gao M, Yu N, Zhang Q, Mou A, Liu Y. 2022. Microbial co-occurrence network topological properties link with reactor parameters and reveal importance of low-abundance genera. NPJ Biofilms Microbiomes 8:3. doi:10.1038/s41522-021-00263-y 35039527 PMC8764041

[57] Freundt S. 2021. Emergence in a complex network with two types of directed edges – a numerical investigation. Results in Physics 30:104819. doi:10.1016/j.rinp.2021.104819

[58] Gao D, Wang S, Wei F, Wu X, Zhou S, Wang L, Li Z, Chen P, Fu B. 2022. The vulnerability of ecosystem structure in the semi-arid area revealed by the functional trait networks. Ecol Indic 139:108894. doi:10.1016/j.ecolind.2022.108894

[59] Ma W, Yang Z, Liang L, Ma Q, Wang G, Zhao T. 2021. Characteristics of the fungal communities and co-occurrence networks in hazelnut tree root endospheres and rhizosphere soil. Front Plant Sci 12:749871. doi:10.3389/fpls.2021.749871 34956257 PMC8692873

[60] Huang L, Guo L. 2007. [Secondary metabolites accumulating and geoherbs formation under enviromental stress]. Zhongguo Zhong Yao Za Zhi 32:277–280. 17455455

[61] Li M, Hou X, Zhang X, Wang Y. 2019. [Construction of database of growth environment of Dao-di herbs]. Zhongguo Zhong Yao Za Zhi 44:3010–3014. doi:10.19540/j.cnki.cjcmm.20190504.104 31602847

[62] Mahajan M, Kuiry R, Pal PK. 2020. Understanding the consequence of environmental stress for accumulation of secondary metabolites in medicinal and aromatic plants. J Appl Res Med Aromat Plants 18:100255. doi:10.1016/j.jarmap.2020.100255

[63] Abegaz BM, Kinfe HH. 2019. Secondary metabolites, their structural diversity, bioactivity, and ecological functions: an overview. Phys Sci Rev 4:12. doi:10.1515/psr-2018-0100

[64] Pajares S, Campo J, Bohannan BJM, Etchevers JD. 2018. Environmental controls on soil microbial communities in a seasonally dry tropical forest. Appl Environ Microbiol 84:e00342-18. doi:10.1128/AEM.00342-18 29959251 PMC6102984

[65] Tang X, He Y, Zhang Z, Wu H, He L, Jiang J, Meng W, Huang Z, Xiong F, Liu J, Zhong R, Han Z, Wan S, Tang R. 2022. Beneficial shift of rhizosphere soil nutrients and metabolites under a sugarcane/peanut intercropping system. Front Plant Sci 13:1018727. doi:10.3389/fpls.2022.1018727 36531399 PMC9757493

[66] Liu F, Zhao Z, Li Z, Gao F, Wang G, Zhou G, Nie J, Peng Y. 2011. [Nutrient balance between N, P, and K in flue-cured tobacco production under different preceding crops planting]. Ying Yong Sheng Tai Xue Bao 22:2622–2626. 22263467

[67] Gao G, Gao Q, Bao M, Xu J, Li X. 2019. Nitrogen availability modulates the effects of ocean acidification on biomass yield and food quality of a marine crop Pyropia yezoensis. Food Chem 271:623–629. doi:10.1016/j.foodchem.2018.07.090 30236725

[68] Jones OAH, Sdepanian S, Lofts S, Svendsen C, Spurgeon DJ, Maguire ML, Griffin JL. 2014. Metabolomic analysis of soil communities can be used for pollution assessment. Environ Toxicol Chem 33:61–64. doi:10.1002/etc.2418 24122881

[69] Song Y, Li XN, Yao S, Yang XL, Jiang X. 2020. Correlations between soil metabolomics and bacterial community structures in the pepper rhizosphere under plastic greenhouse cultivation. Sci Total Environ 728:138439. doi:10.1016/j.scitotenv.2020.138439 32361108

[70] Bi BY, Wang K, Zhang H, Wang Y, Fei HY, Pan RP, Han FP. 2021. Plants use rhizosphere metabolites to regulate soil microbial diversity. Land Degrad Dev 32:5267–5280. doi:10.1002/ldr.4107

[71] Bao Y-F, Li J-Y, Zheng L-F, Li H-Y. 2015. Antioxidant activities of cold-nature Tibetan herbs are signifcantly greater than hot-nature ones and are associated with their levels of total phenolic components. Chin J Nat Med 13:609–617. doi:10.1016/S1875-5364(15)30057-1 26253494

[72] Jackson ML. 1959. Soil chemical analysis, p 498. Prentice Hall of India Pvt. Ltd., New Delhi.

[73] Nelson DW, Sommers LE. 1996. Total carbon, organic carbon, and organic matter, p 539–579. In Sparks AL, Page PA, Helmke RH, Loeppert PN, Soltanpour MA, Tabatabai CT, Sumner ME (ed), Methods of soil analysis. Part 3. Chemical methods. American society of agronomy, Wisconsin.

[74] Brookes PC, Landman A, Pruden G, Jenkinson DS. 1985. Chloroform fumigation and the release of soil nitrogen: a rapid direct extraction method to measure microbial biomass nitrogen in soil. Soil Biol Biochem 17:837–842. doi:10.1016/0038-0717(85)90144-0

[75] Vance ED, Brookes PC, Jenkinson DS. 1987. An extraction method for measuring soil microbial biomass C. Soil Biol Biochem 19:703–707. doi:10.1016/0038-0717(87)90052-6

[76] Chen B, Yang H, Song W, Liu C, Xu J, Zhao W, Zhou Z. 2016. Effect of N fertilization rate on soil alkali-hydrolyzable N, subtending leaf N concentration, fiber yield, and quality of cotton. Crop J 4:323–330. doi:10.1016/j.cj.2016.03.006

[77] Baran A, Mierzwa-Hersztek M, Gondek K, Tarnawski M, Szara M, Gorczyca O, Koniarz T. 2019. The influence of the quantity and quality of sediment organic matter on the potential mobility and toxicity of trace elements in bottom sediment. Environ Geochem Health 41:2893–2910. doi:10.1007/s10653-019-00359-7 31236855 PMC6856041

[78] Koistinen J, Sjöblom M, Spilling K. 2019. Total nitrogen determination by a spectrophotometric method, p 81–86. In Spilling K (ed), Biofuels from algae. Methods in molecular biology. Humana, New York, NY. 10.1007/7651_2019_206 30734162

[79] Dodor D, Tabatabai M. 2020. Alkaline hydrolyzable organic nitrogen as an index of nitrogen mineralization in soils: relationship with activities of arylamidase and amidohydrolases. Commun Soil Sci Plant Anal 51:1757–1766. doi:10.1080/00103624.2020.1791162

[80] Tian H, Qiao J, Zhu Y, Jia X, Shao M. 2021. Vertical distribution of soil available phosphorus and soil available potassium in the critical zone on the Loess Plateau, China. Sci Rep 11:3159. doi:10.1038/s41598-021-82677-4 33542419 PMC7862489

[81] Garcia-Elias A, Alloza L, Puigdecanet E, Nonell L, Tajes M, Curado J, Enjuanes C, Díaz O, Bruguera J, Martí-Almor J, Comín-Colet J, Benito B. 2017. Defining quantification methods and optimizing protocols for microarray hybridization of circulating microRNAs. Sci Rep 7:7725. doi:10.1038/s41598-017-08134-3 28798363 PMC5552704

[82] Wu L, Wen C, Qin Y, Yin H, Tu Q, Van Nostrand JD, Yuan T, Yuan M, Deng Y, Zhou J. 2015. Phasing amplicon sequencing on Illumina Miseq for robust environmental microbial community analysis. BMC Microbiol 15:125. doi:10.1186/s12866-015-0450-4 26084274 PMC4472414

[83] Zhang J, Kobert K, Flouri T, Stamatakis A. 2014. PEAR: a fast and accurate Illumina paired-End reAd mergeR. Bioinformatics 30:614–620. doi:10.1093/bioinformatics/btt593 24142950 PMC3933873

[84] Schmieder R, Edwards R. 2011. Quality control and preprocessing of metagenomic datasets. Bioinformatics 27:863–864. doi:10.1093/bioinformatics/btr026 21278185 PMC3051327

[85] Martin M. 2011. CUTADAPT removes adapter sequences from high-throughput sequencing reads. EMBnet J 17:10–12. doi:10.14806/ej.17.1.200

[86] Edgar RC. 2013. UPARSE: highly accurate OTU sequences from microbial amplicon reads. Nat Methods 10:996–998. doi:10.1038/nmeth.2604 23955772

[87] Edgar RC. 2016. SINTAX: a simple non-Bayesian taxonomy classifier for 16S and ITS sequences. bioRxiv. doi:10.1101/074161

[88] Caporaso JG, Kuczynski J, Stombaugh J, Bittinger K, Bushman FD, Costello EK, Fierer N, Peña AG, Goodrich JK, Gordon JI, et al.. 2010. QIIME allows analysis of high-throughput community sequencing data. Nat Methods 7:335–336. doi:10.1038/nmeth.f.303 20383131 PMC3156573

[89] Wang Q, Garrity GM, Tiedje JM, Cole JR. 2007. Naive Bayesian classifier for rapid assignment of rRNA sequences into the new bacterial taxonomy. Appl Environ Microbiol 73:5261–5267. doi:10.1128/AEM.00062-07 17586664 PMC1950982

[90] Elhaik E. 2022. Principal component analyses (PCA)-based findings in population genetic studies are highly biased and must be reevaluated. Sci Rep 12:14683. doi:10.1038/s41598-022-14395-4 36038559 PMC9424212

[91] Segata N, Izard J, Waldron L, Gevers D, Miropolsky L, Garrett WS, Huttenhower C. 2011. Metagenomic biomarker discovery and explanation. Genome Biol 12:R60. doi:10.1186/gb-2011-12-6-r60 21702898 PMC3218848

[92] Huang W, Sun D, Chen L, An Y. 2021. Integrative analysis of the microbiome and metabolome in understanding the causes of sugarcane bitterness. Sci Rep 11:6024. doi:10.1038/s41598-021-85433-w 33727648 PMC7966368

[93] Xu H, Huang Y, Xiong X, Zhu H, Lin J, Shi J, Tang C, Xu J. 2023. Changes in soil Cd contents and microbial communities following Cd-containing straw return. Environ Pollut 330:121753. doi:10.1016/j.envpol.2023.121753 37127235

[94] Yu F-M, Jayawardena RS, Thongklang N, Lv M-L, Zhu X-T, Zhao Q. 2022. Morel production associated with soil nitrogen-fixing and nitrifying microorganisms. J Fungi (Basel) 8:299. doi:10.3390/jof8030299 35330300 PMC8950353

[95] Cohen I, Huang Y, Chen J, Benesty J. 2009. Pearson correlation coefficient, p 1–4. In Noise reduction in speech processing. Springer, Berlin, Heidelberg.

[96] Hollander M, A. Wolfe D, Chicken E. 2015. Nonparametric statistical methods, p 121–162. In Daryl SP (ed), Biostatistics and microbiology: a survival manual. Springer, New York, NY.

